# Pulmonary Thromboembolism Complicating Amebic Liver Abscess: First Reported Case in the United States—Case Report and Literature Review

**DOI:** 10.1155/2015/516974

**Published:** 2015-07-30

**Authors:** Devon McKenzie, Michael Gale, Sunny Patel, Grazyna Kaluta

**Affiliations:** ^1^Department of Internal Medicine, New York University School of Medicine, Woodhull Medical and Mental Health Center, Brooklyn, NY 11206, USA; ^2^Department of Infectious Disease, Department of Medicine, New York University School of Medicine, Woodhull Medical and Mental Health Center, Brooklyn, NY 11206, USA

## Abstract

Even in the absence of Amebic colitis, Amebic liver abscess (ALA) is the most common extraintestinal complication of *Entamoeba histolytica* infection. In the USA, it is most prevalent in middle aged immigrant males from endemic countries such as Africa, Mexico, and India. One of the complications of ALA is inferior vena cava (IVC) thrombosis, which is believed to result from the mechanical compression of the IVC and the consequent thrombogenic nidus elicited from the resultant inflammatory response. There are very few reported cases and even fewer in which the thrombus became a harbinger to pulmonary thromboembolism. We present the case of a 43-year-old male from West Africa who presented with the chief complaint of right upper quadrant abdominal pain for one week associated with persistent nonproductive cough. He had a positive serum *Entamoeba histolytica* antibody with CT scan findings of a hepatic abscess with thrombosis of the hepatic vein and inferior vena cava and numerous bilateral pulmonary emboli. This amebic liver abscess was successfully treated with metronidazole and paromomycin, whereas the pulmonary thromboembolism was managed with medical anticoagulation. Based on current knowledge, this is the first reported case in the USA.

## 1. Introduction

Amebic liver abscess is the most common extraintestinal manifestation of amebiasis. Although there is no current data as to its incidence/prevalence in the US partly because it is no longer a notifiable disease, it has its highest prevalence in immigrant men from endemic countries who are in the fourth to fifth decade of life. One of the rare complications of this disease is inferior vena cava thrombosis of which there are very few reported cases. As to current knowledge, however, there are no cases reported of this inferior vena cava thrombosis proceeding to overt pulmonary embolism in the United States. Hence, this case of 43-year-old West African male who was accurately diagnosed and effectively treated will be the first.

## 2. Case Report

We present the case of a 43-year-old West African male with a past medical history of untreated hepatitis B and chronic alcohol abuse. He complained of a one-week history of fever, chills, and worsening right upper quadrant abdominal pain with nausea and nonbloody, billous vomiting. The pain was described as being dull in nature, nonradiating, exacerbated by deep inspiration, and 8/10 in severity on the numeric rating scale. He also mentioned having a progressive and persistent nonproductive cough of the same duration. These symptoms began three weeks after he returned from a one-month vacation in West Africa.

On presentation, he was febrile with a temperature of 100.8 F, his heart rate was 108 beats per minute and he had tachypnea of 25 breaths per minute. Physical examination of the abdomen was significant for a persistent diffuse abdominal pain, most pronounced in the right hypochondrium, which hindered effective palpation of the liver borders. Exam of the thorax was significant for rapid shallow breathing with resultant decreased breath sounds diffusely, most pronounced over the right lung base. Hematological investigation was significant for a WBC of 30,500/*μ*L (4500–11000/*μ*L) with 80% polymorphonuclear leukocyte count (57–67%). AST/ALT was mildly elevated at 70/51 U/L (7–40 U/L) with an albumin of 2.7 g/dL (3.5–5.5 g/dL) and mildly elevated prothrombin time and INR of 15.4 s (11–15 s) and 1.42 s (0.8–1.2 s), respectively. Additionally, his PTT was also elevated at 36.7 s (20–35 s) and his D-dimer was elevated at 5.7 mg/L (0.2–0.7 mg/L). Of note, the bilirubin and creatinine were within normal limits.

**Figure 1:**
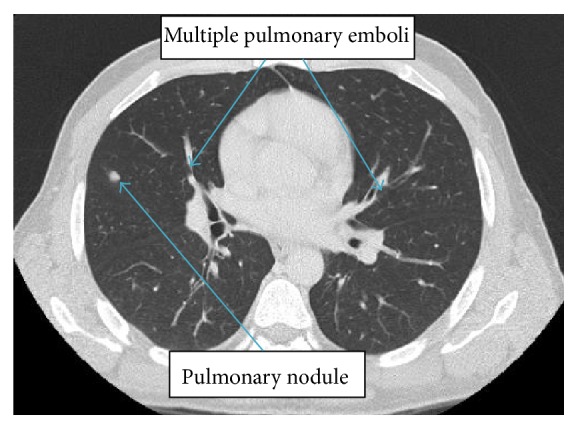
Bilateral pulmonary emboli with pulmonary nodule.

**Figure 2:**
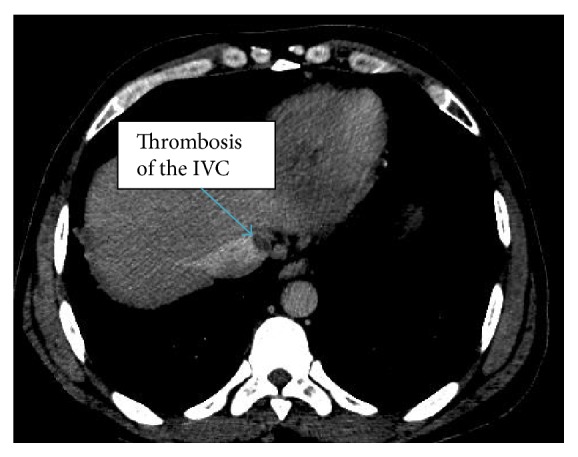
CT scan showing thrombosis of the hepatic portion of the IVC.

**Figure 3:**
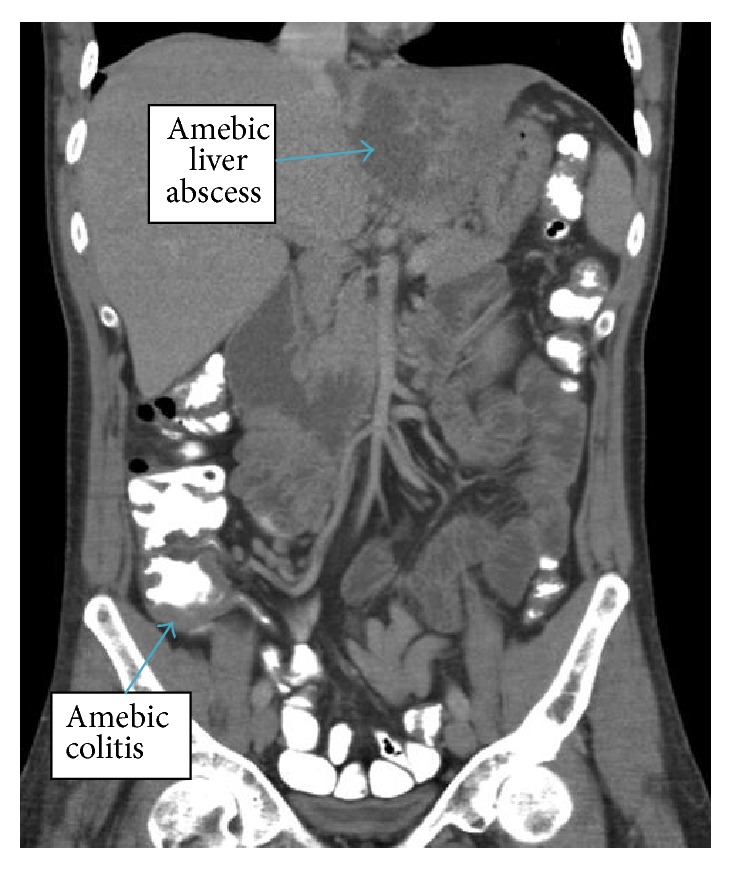
CT abdomen showing 8.5 × 9.6 × 6.8 cm liver mass and cecal wall thickening suggestive of colitis.

Due to positive D-dimer, tachypnea, tachycardia, and high suspicion of pulmonary embolism (Wells score of 4.5), given recent long distance travel, CT scans of the chest and abdomen with contrast were done. It found multiple left lower lobe subsegmental, right lower lobe, and right upper lobe segmental pulmonary emboli. There were also four small, scattered nonspecific pulmonary nodules. There was a 7 mm noncalcified lateral anterior right upper lobe nodule. There were pleural-based anterior bilateral upper lobe noncalcified nodules measuring up to 4 mm on the right and there was also a 3 mm lateral basal right lower lobe noncalcified nodule as well as a posterior left apical pleural-based 3 mm noncalcified nodule (Figure 1). Also discovered were thrombosis of the hepatic portion of the IVC and left hepatic vein (Figure 2) and a large, irregular, heterogeneous marginated mass in the left hepatic lobe with a complex appearance and extensive enhancing septa, measuring approximately 8.5 × 9.6 × 6.8 cm. This appearance was highly suggestive of an abscess in the presence of concurrent CT evidence of colitis (Figure 3). Transthoracic echocardiogram was only significant for moderate tricuspid regurgitation with a pulmonary arterial pressure of 39 mmHg but it was negative for intracardiac thromboembolism.

At this time, amebic liver abscess was the leading differential; hence, he was admitted and started on metronidazole 500 mg intravenously every eight hours along with warfarin bridged with unfractionated heparin for pulmonary emboli.

He remained hospitalized for twelve days, during which time serum antibody for* Entameba histolytica* was positive (confirming diagnosis) and AFP and CEA were found to be within normal limits ruling out possible malignancy. Work-up for underlying hypercoagulable states including factor V leiden, protein C, and protein S were all within normal limits and lower extremity duplex scan found no evidence of deep vein thrombosis. Since the clinical picture was highly suggestive for ALA and the patient was responding well, we believed further confirmatory test were unnecessary as it would not have changed our management. He finished a ten-day course of metronidazole with gradual amelioration of his fever, abdominal pain, and normalization of liver enzymes. He was subsequently discharged on paromomycin for seven days and continued on warfarin to follow as an outpatient.

## 3. Discussion

Even in the absence of amebic colitis, amebic liver abscess (ALA) is the most common extraintestinal manifestation of* Entamoeba histolytica* infection. ALA results from the ascension of the* Entamoeba histolytica* protozoa from the colon through the portal venous system and the consequent establishment of a hepatic infection. The most prevalent group in the US is migrant middle aged males from endemic places such Africa, Mexico, India, and parts of Central and South America. Although the reason for this is not fully understood, it is suggested that hormonal effects and previous hepatocellular damage create a nidus for portal seeding [1]. Hence, ALA should be suspected in any patient who has travelled to endemic areas, presenting with fever, right upper quadrant pain, and hepatic tenderness [2].

Diagnosis is normally made with a combination of serologic and imaging studies. In regard to the latter, computed tomography is ideal because it is the best choice when there is suspected thromboembolism. The diagnosis can then be confirmed with* E. histolytica* antibodies which are detectable in 92% to 97% of patients at time of presentation [1].

IVC thrombosis is an infrequent complication of ALA [2]. It is believed to develop secondary to the mechanical compression of the IVC by the abscess which interrupts the laminar blood flow and the consequent thrombogenic nidus elicited from the resultant inflammatory response [3]. There have been very few reported cases of this complication. For instance, in 1981 Huddle [4] reported a case of a 26-year-old south African male with associated nephrotic syndrome. In 2008, Sodhi et al. [5] reported a case of a 57-year-old male in India. In 2011, 3 cases in Korea were reported by Sarda et al. [6] and, in 2012, another case of a 47-year-old male was reported in India by Ray et al. [3]. There was even a pediatric case complicated with right atrial thrombus that was reported by Siddiqui et al. in India in 2013 [7]. However, in all the reported cases, there was no documented evidence of pulmonary embolism until May 2014 when Thati published the first reported case in India [8]. Yet, none has been documented within the United States, making this incidence unique, because, unlike the former, the United States is not an endemic area for* Entamoeba histolytica.*

In treating this patient, the ALA and the pulmonary emboli were treated concurrently with great response. For the ALA, metronidazole was used as the tissue agent against the live protozoa followed by paromomycin as a luminal agent to prevent further infection by eliminating intraluminal cyst. The latter achieved this by specifically inhibiting protozoan protein synthesis through binding the 30S ribosomal RNA in the aminoacyl-tRNA site, causing misreading of mRNA codons [9, 10]. The PE was treated with anticoagulation therapy (heparin bridged to warfarin).

As stated earlier, this is the first reported case of IVC thrombosis secondary to ALA progressing to PE in the United States. The importance of this case and its ilk is that they raise the question of whether there was covert asymptomatic pulmonary embolism in the other reported cases of IVC thrombosis. It also begs the question of whether there is any benefit to investigate for pulmonary embolism in patients presenting with IVC thrombosis in the setting of ALA.

## Abbreviations

ALT: Alanine transaminase
ALA: Amebic liver abscess
AST: Aspartate transaminase
PE: Pulmonary embolism
IVC: Inferior vena cava
INR: International normalized ratio
PT: Prothrombin Time.
